# Influence of the foveal curvature on myopic macular complications

**DOI:** 10.1038/s41598-019-53443-4

**Published:** 2019-11-15

**Authors:** Un Chul Park, Dae Joong Ma, Woon Hyung Ghim, Hyeong Gon Yu

**Affiliations:** 1 Department of Ophthalmology, Seoul National University College of Medicine, Seoul, Korea; 2 Retinal Degeneration Research Laboratory, Seoul National University Hospital Biomedical Research Institute, Seoul, Korea

**Keywords:** Retina, Retinal diseases

## Abstract

In this cross-sectional study, we investigated whether a foveal curvature affects the development of two major myopic macular complications, myopic traction maculopathy (MTM) and myopic choroidal neovascularization (mCNV). In high myopic eyes (axial length ≥ 26.5 mm, refractive error ≤ −6 diopters) with posterior staphyloma, three different parameters of foveal curvature (staphyloma height, coefficient *a*, and curvature index) calculated based on the retinal pigment epithelium hyperreflective line in spectral domain optical coherence tomography image were compared among the MTM (72 eyes), mCNV (58 eyes), and control (69 eyes) group. The three curvature parameters showed a significant correlation with each other (all *P*’s < 0.001). The axial length, refractive error, and staphyloma types were comparable among the groups, but the means of all three curvature parameters were significantly greater in the MTM group compared to the mCNV and control groups (all *P*’s < 0.001). Furthermore, the curvature parameters had a significant correlation with myopic severity in the MTM group, but not in the other groups. These results suggest that a steeper change of foveal curvature plays a role in the development of MTM but not mCNV in high myopes.

## Introduction

Myopia is one of the leading causes of visual impairment worldwide^1^. Pathologic myopia (PM) usually refers to a severe form of myopia resulting in the loss of visual acuity. The definition of PM has been inconsistent among studies, but the current concept of PM is based on the presence of myopic maculopathy rather than the degree of myopia^2,3^. Recently, a meta-analysis for PM (META-PM) study group suggested a new, simplified PM classification system based on the severity of chorioretinal atrophy and the presence of three “plus” lesions (lacquer crack, choroidal neovascularization, and Fuchs spot) strongly associated with central vision loss^4^.

Convex configuration of the sclera within the area of the posterior staphyloma results in stretching of the sensory retina, retinal pigment epithelium (RPE), and choroid, thus causing mechanical damage. Compared to high myopic eyes without posterior staphyloma, macular complications that can influence the central vision, including chorioretinal atrophy, myopic choroidal neovascularization (mCNV), and myopic traction maculopathy (MTM), are found more frequently in eyes with posterior staphyloma^5^.

However, association with the severity of staphyloma could be different among the forms of myopic macular complications. For example, the incidence of macular retinoschisis has been reported to be higher among patients with more severe staphyloma^6^, while mCNV was more frequent in the shallow form of staphyloma^7^. Although the grading of staphyloma was based only on stereoscopic ophthalmoscopy and ultrasound findings, these results suggest that the shape of posterior staphyloma could be a determinant in the development of different types of myopic macular complication.

The optical coherence tomography (OCT) provides detailed structural image of the posterior pole and can be useful tool for quantitative assessment of retinal configuration around the fovea. The curvature of the inner surface of the posterior staphyloma measured on OCT images has a potential for use as an imaging biomarker in the development of vision-threatening macular complications in patients with PM and associated clinical conditions, allowing the delineation of pathogeneses and the development of treatment approaches. In this study, we investigated whether the presence of the two major myopic complications involving macula, the MTM and mCNV, has association with the foveal curvature in the spectral domain OCT images in high myopic eyes with posterior staphyloma. We also evaluated the clinical significance of the curvature index as a new method to determine foveal curvature.

## Results

In total, 72 eyes (72 patients) with MTM, 58 eyes (58 patients) with mCNV, and 69 myopic control eyes (69 patients) were included in this study. The baseline features among the three groups were comparable except for age and best-corrected visual acuity (BCVA) (Table 1). In the control group, the mean age was significantly lower and the mean BCVA was significantly better than the MTM or mCNV group. The mean age and BCVA were comparable between the MTM and mCNV group. Myopia severity including the axial length (29.8 ± 1.7 mm vs. 30.0 ± 1.4 mm vs. 29.5 ± 1.5 mm for the MTM, mCNV, and control groups, respectively; *P* = 0.184), refractive error (−12.6 ± 3.7 D vs. −12.4 ± 2.7 D vs. −11.9 ± 2.8 D; *P* = 0.282) were comparable among the groups. Also, the distribution of staphyloma types and myopic macular degenerative changes were also comparable among groups.

**Table 1:** Patients demographics and baseline characteristics.

Parameters	MTM group (72 eyes)	mCNV group (58 eyes)	Control group (69 eyes)	*P* – value
Age (years)	60.7 ± 9.1	60.0 ± 10.6	52.3 ± 9.1	<0.001*
Gender (M/F)	17/55 (76.4%)	11/47 (81.0%)	20/49 (71.0%)	0.418**
BCVA (logMAR, by eyes)	0.73 ± 0.54	0.80 ± 0.39	0.54 ± 0.41	0.003*
Axial length (mm)	29.8 ± 1.7	30.0 ± 1.4	29.5 ± 1.5	0.184*
Refractory error (Diopter)^†^	−12.6 ± 3.7	−12.4 ± 2.7	−11.9 ± 2.8	0.282*
Type of posterior staphyloma				0.592**
Wide, macular	31 (43.1%)	29 (50.0%)	28 (40.6%)	
Narrow, macular	36 (50.0%)	27 (46.6%)	39 (56.5%)	
Others	5 (6.9%)	2 (3.4%)	2 (2.9%)	
Myopic macular degenerative change				0.582**
Tessellated fundus (category 1)	8 (11.1%)	5 (8.6%)	12 (17.4%)	
Diffuse chorioretinal atrophy (category 2)	34 (47.2%)	27 (46.6%)	33 (47.8%)	
Patchy chorioretinal atrophy (category 3)	29 (40.3%)	26 (44.8%)	24 (34.8%)	
Macular atrophy (category 4)	1 (1.4%)	0 (0%)	0 (0%)	
Subfoveal choroidal thickness (µm)	39.1 ± 19.7	34.0 ± 17.8	37.9 ± 18.5	0.281*

*One-way analysis of variance.

**chi-square test.

^†^Excluded pseudophakic eyes (10 eyes in MTM, 6 eyes in mCNV group, 5 eyes in control group).

MTM = Myopic tractional maculopathy; mCNV = myopic choroidal neovascularization; BCVA = Best-corrected visual acuity; logMAR = logarithm of minimal angle of resolution.

**Figure 1:**
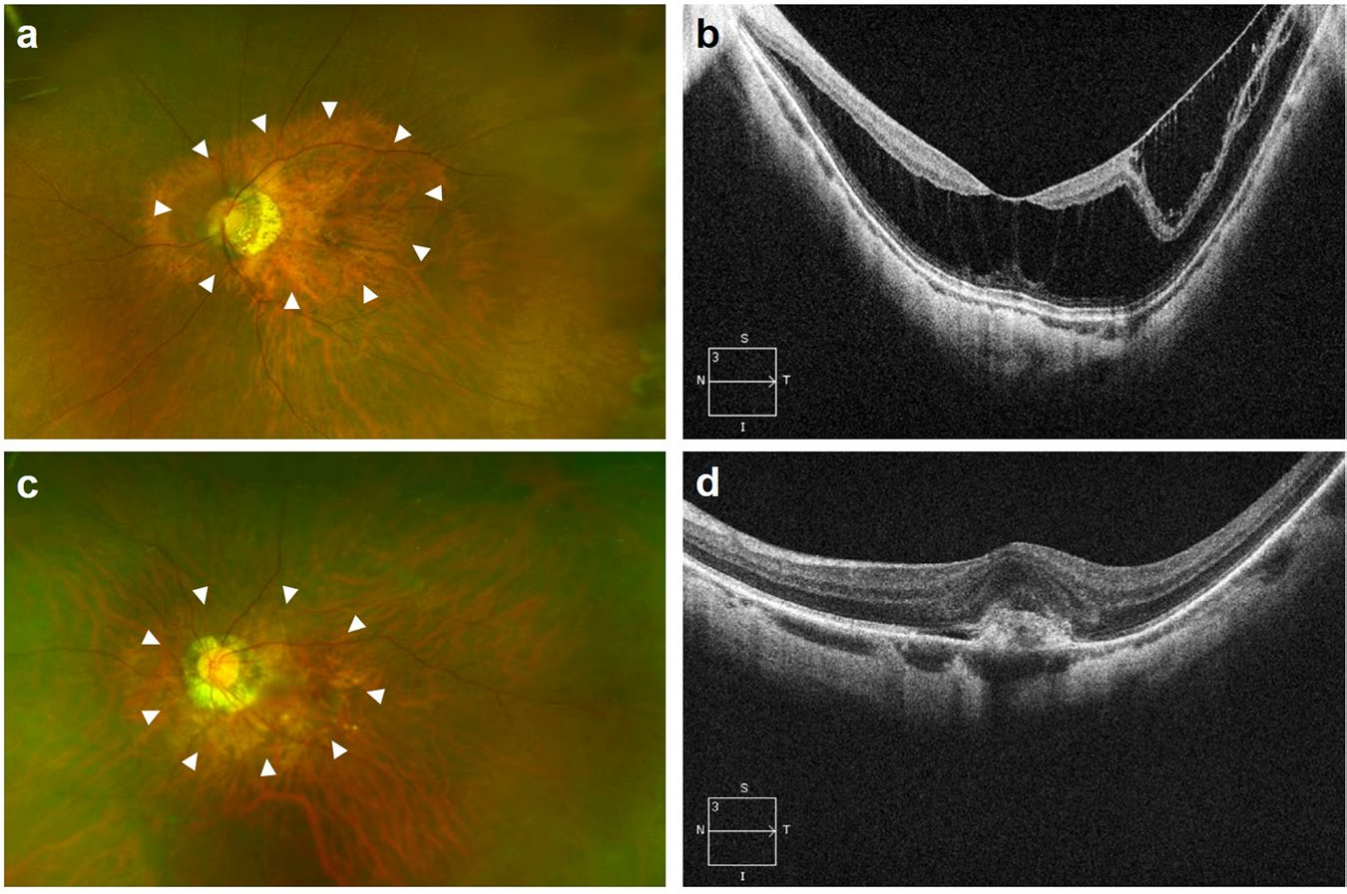
Representative wide-field retinal images and optical coherence tomography B-scan images of patients with myopic traction maculopathy and myopic choroidal neovascularization (mCNV). (**a**,**b**) A 50-year-old female with foveoschisis (axial length, 29.1 mm; refractive error, −12.75 D). Wide, macular staphyloma is visible (arrowhead). Her foveal curvature parameters were as follows: total staphyloma height, 3,028 μm; coefficient *a*, 4.78 × 10^−4^; and curvature index, 1.066. (**c**,**d**) A 61-year-old male with mCNV (axial length, 29.9 mm; refractive error, −12.25 D). Wide, macular staphyloma is visible (arrowhead). Despite comparable axial length and refractive error, his curvature parameters were smaller: total staphyloma height, 1,696 μm; coefficient *a*, 3.40 × 10^−4^; and curvature index, 1.031.

There was a significant difference among the three groups in all curvature parameters including the total staphyloma height (2,614.0 ± 944.3 vs. 2,030.7 ± 879.6 μm vs. 2,042.2 ± 771.1 μm; *P* < 0.001), average coefficient *a* × 10^4^ (4.33 ± 2.35 vs. 3.11 ± 1.39 vs. 3.14 ± 1.56; *P* < 0.001), and average curvature index (1.066 ± 0.048 vs. 1.044 ± 0.027 vs. 1.042 ± 0.021; *P* < 0.001). For all parameters, the MTM group showed significantly greater foveal curvature than the mCNV group (*P* < 0.001) and the control group (*P* < 0.001). However, no differences in any of the curvature parameters were observed between the mCNV and control groups (all *P*’s = 1.000). Representative cases of MTM and mCNV are shown in Fig. 1. For each curvature parameter, the horizontal and vertical values also showed the same pattern of difference, whereby the MTM group had a significantly greater curvature than the mCNV and control group (Table 2). However, for each staphyloma height, no significant difference was found among the three groups in the temporal and inferior heights.

**Table 2:** Comparison of curvature parameters between the patients with myopic traction maculopathy group and myopic choroidal neovascularization.

		MTM group (72 eyes)	mCNV group (58 eyes)	Control group (69 eyes)	*P* – value*
Staphyloma height (µm)	Nasal (H_N_)	599.3 ± 434.0	257.2 ± 467.9	382.8 ± 347.0	<0.001
	Temporal (H_T_)	727.0 ± 401.6	736.7 ± 357.4	627.4 ± 298.1	0.147
	Superior (H_S_)	852.8 ± 417.6	658.9 ± 397.6	597.2 ± 347.7	<0.001
	Inferior (H_I_)	434.8 ± 427.3	377.9 ± 347.8	434.9 ± 296.9	0.610
	Horizontal (H_N_ + H_T_)	1326.4 ± 593.2	993.9 ± 503.2	1010.2 ± 446.0	0.001
	Vertical (H_S_ + H_I_)	1287.5 ± 448.2	1036.8 ± 459.2	1032.0 ± 415.7	<0.001
	Total (H_N_ + H_T_ + H_S_ + H_I_)	2614.0 ± 944.3	2030.7 ± 879.6	2042.2 ± 771.1	<0.001
Coefficient *a* × 10^4^	Horizontal	4.47 ± 2.54	2.96 ± 1.46	3.21 ± 1.62	<0.001
	Vertical	4.21 ± 2.37	3.25 ± 1.48	3.07 ± 1.61	0.001
	Average	4.33 ± 2.35	3.11 ± 1.39	3.14 ± 1.56	<0.001
Curvature index	Horizontal	1.064 ± 0.053	1.044 ± 0.029	1.043 ± 0.024	0.002
	Vertical	1.069 ± 0.050	1.043 ± 0.031	1.041 ± 0.022	<0.001
	Average	1.066 ± 0.048	1.044 ± 0.027	1.042 ± 0.021	<0.001

*One-way analysis of variance.

MTM = Myopic tractional maculopathy; mCNV = myopic choroidal neovascularization.

In the MTM group, the foveal curvature parameters were significantly correlated with the severity of myopia, but there was no relationship between the total staphyloma height and refractive error; patients with steeper foveal curvature were more likely to have greater axial length and more myopic refractive error (Table 3). However, the curvature parameters did not show a significant correlation with the severity of myopia in the mCNV and the control group except between the total staphyloma height and refractive error. The severity of myopic macular complication, i.e., the foveoschisis height (FSH) in the MTM group and the CNV size in the mCNV group, were not correlated with any of the foveal curvature parameters.

**Table 3:** Correlation of curvature parameters with axial length, refractive error, and severity parameters in the myopic traction maculopathy, myopic choroidal neovascularization, and control group.

		Total staphyloma height		Coefficient *a*		Curvature index	
		*r*	*P* – value	*r*	*P* – value	*r*	*P* – value
MTM group	Axial length	0.300	0.010	0.358	0.002	0.417	<0.001
	Refractive error*	−0.226	0.078	−0.397	0.001	−0.258	0.043
	Foveoschsis height	0.048	0.689	−0.088	0.461	−0.025	0.835
mCNV group	Axial length	−0.130	0.330	−0.054	0.686	−0.146	0.275
	Refractive error*	0.314	0.023	0.148	0.294	0.156	0.271
	CNV size	0.079	0.527	−0.022	0.864	0.061	0.629
Control group	Axial length	0.027	0.825	0.053	0.666	0.131	0.285
	Refractive error*	−0.096	0.452	−0.157	0.216	−0.063	0.622

MTM = Myopic tractional maculopathy; mCNV = myopic choroidal neovascularization.

*Excluded pseudophakic eyes (10 eyes in MTM, 6 eyes in mCNV group, 5 eyes in control group).

**Figure 2:**
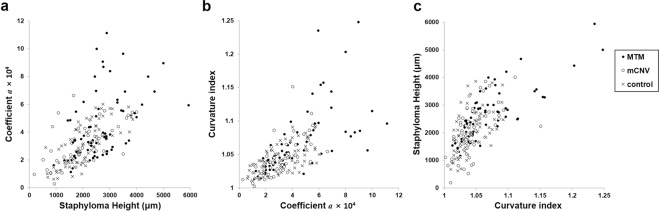
Scatterograms depicting the correlations among the different methods for staphyloma curvature measurement. (**a**) Staphyloma height *vs*. coefficient *a*. (**b**) Coefficient *a vs*. curvature index. (**c**) Curvature index *vs*. staphyloma height. All comparisons showed a significant positive correlation (*P* < 0.001). MTM, Myopic tractional maculopathy; mCNV, myopic choroidal neovascularization.

There was a significant correlation among the three foveal curvature parameters, namely between the total staphyloma height and the average coefficient *a*, between the average coefficient *a* and the average curvature index, and between the average curvature index and the total staphyloma height (*r* = 0.626, *r* = 0.670, and *r* = 0.700, respectively; all *P*’s < 0.001; Fig. 2).

## Discussion

In this study, the MTM, mCNV, and control group showed comparably high axial length and refractive errors, but the foveal curvature, which represents the steepness of the posterior staphyloma, was significantly greater in the MTM group than in other group. This suggests that mechanical pathogeneses underlying the MTM might be different from mCNV or other myopic maculopathies.

There has been no standard method to assess the curvature of posterior staphyloma to date. The morphological assessment of the eyeball shape in highly myopic patients has been based so far on ultrasonography and, more recently, three-dimensional magnetic resonance imaging (MRI)^6,8,9^. The three-dimensional MRI can visualize the entire ocular shape *in situ*, but its usefulness in highly myopic eyes seems to be limited to the qualitative categorization of the eyeball shape^8,9^. On the other hand, recent advances in OCT enabled the detection of pathological changes in myopic maculopathies as well as the quantitative assessment of posterior pole shape in high myopes^10,11^.

In this study, we adopted two known applicable methods using the hyperreflective RPE lines in the B-scan image of spectral domain OCT as representative lines of the foveal curvature within staphyloma. A coefficient *a* can be obtained after fitting the tomographic image to a second-order polynomial equation (*ax*^2^ + *bx* + *c*), and represents the steepness of the staphyloma curvature. Baba *et al*.^12^ reported a decrease in coefficient *a* and resolution of schitic change and retinal detachment after scleral imbrication combined with vitrectomy in high myopic patients with myopic retinoschisis, suggesting the influence of staphyloma curvature in the pathogenesis of myopic retinoschisis. A staphyloma height, which represents the angle of the posterior staphyloma in relation to the horizontal plane, is another easy method to quantify the shape of posterior staphyloma. Increased height of staphyloma has been reported to be associated with myopic foveoschisis and mCNV^13,14^, but its difference among the types of myopic macular complication has not been investigated. In the present study, we adopted a novel parameter, the curvature index, as the third method to quantify the foveal curvature within posterior staphyloma. This index showed strong correlations with the staphyloma height and coefficient *a*, suggesting that it can be used as another useful anatomic indicator of the steepness of foveal curvature. Curvature index represents the foveal contour better than the other methods, because it is based on the ratio of the elongated RPE line due to posterior bowing to the straight distance. Even between the eyes with same staphyloma heights, curvature index could be different according to the entire shape of RPE line (Supplementary Fig. S1).

All three tested parameters of foveal curvature were greater in the MTM group than in other groups, representing a steeper shape of staphyloma (Table 2). Different foveal curvature may not reflect the differences in the severity of staphyloma because the axial length and distribution of staphyloma types were comparable among the groups. Moreover, in a previous study^6^, prevalence of mCNV and macular retinoschisis was not correlated with the staphyloma types per Curtin classification^15^. This suggests that the location and en face size of the staphyloma are not associated with the type of myopic macular complication, but the foveal curvature may be a relevant factor as indicated by our results.

**Figure 3:**
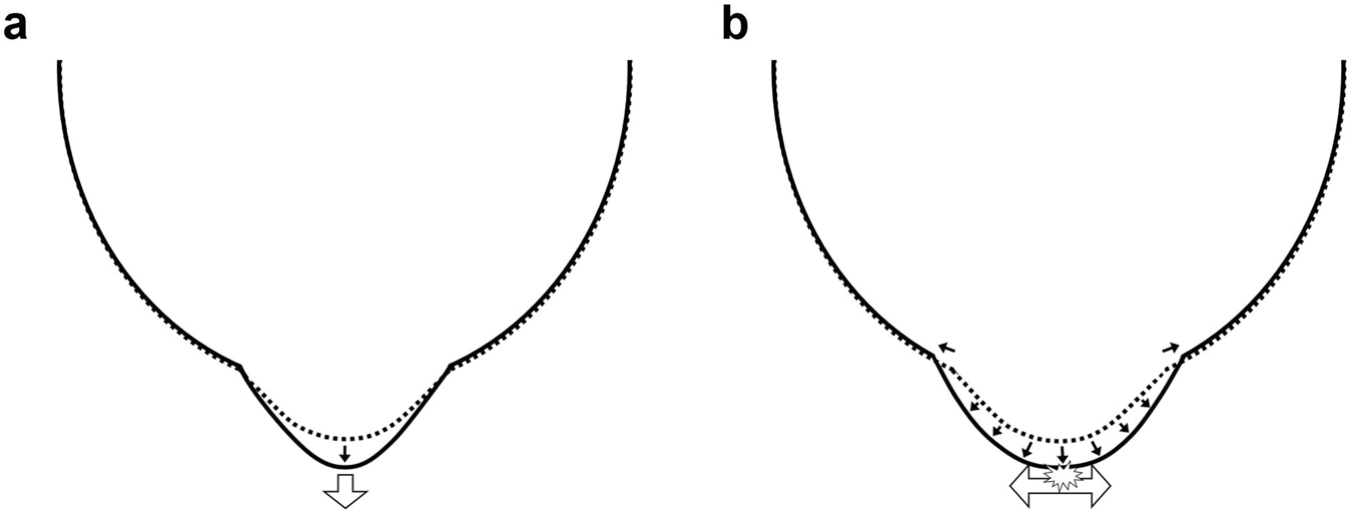
In eyes with posterior staphyloma with a steeper curvature, the posterior pole of the staphyloma is likely to be primarily under anterior-posterior traction, resulting in retinal schitic change and myopic tractional maculopathy (**a**). Expansion of the posterior staphyloma with a shallower curvature might be more multidirectional, and the apex of the staphyloma is likely to be primarily under tangential traction, resulting in a break in the Bruch membrane/retinal pigment epithelium and subsequent formation of choroidal neovascularization (**b**).

Although OCT scan length did not cover the entire extent of posterior staphyloma in this study, extrapolated shape of staphyloma based on the foveal curvature leads to a plausible explanation that the difference in foveal curvature between the MTM and other groups might have resulted from the different directional nature of staphyloma expansion as shown in Fig. 3. Both anterior-posterior and tangential traction forces may be exerted on the apex of the posterior staphyloma due to its expansion, but the sensory retina, RPE, Bruch membrane, and choroid within the staphyloma with steeper curvature are more likely to have been primarily under anterior-posterior traction, resulting in retinoschitic change and development of MTM. However, if the axial length is comparable, staphyloma with a less steep curvature might have undergone more multi-directional expansion around the fovea; those tissues within the staphyloma are likely to be primarily under tangential traction, resulting in a break in the Bruch membrane/RPE and subsequent formation of CNV. The correlation between the foveal curvature and myopic severity only in the MTM group, but not in the mCNV and control group, also supports the notion of a different mechanical pathogenesis of MTM from other myopic maculopathies.

Our results are comparable to a study by Miyake *et al*.^16^, which reconstructed a color topographic map of the posterior pole curvature using Bruch membrane plots in radial OCT scans. When the mean curvature values over the entire scanned area were compared among myopic complications, the eyes with retinoschisis were found to have steeper curvatures than those with mCNV. However, in contrast to the present study, eyes with chorioretinal atrophy showed greatest curvature. The reason for this discrepancy is unclear but may be because the chorioretinal atrophy group in their study consisted of only severe cases with patchy atrophic lesion of >3 disc area and had longer axial length and older age compared to other groups.

The pathogenesis of MTM and mCNV has not been fully elucidated. Recent advancement in OCT imaging suggested intrinsic non-compliance of the internal limiting membrane, tangential traction on the retinal surface by the residual vitreous cortex or epiretinal membrane, and anterior vitreomacular traction associated with posterior elongation of the sclera as the mechanism of MTM^17–19^. Due to the relative tautness of the inner retina, the flexibility to conform to the concavity of the enlarging posterior staphyloma is different between the inner and outer retina, resulting in retinal splitting. In the MTM group, the severity of MTM determined by the height of retinoschisis at the fovea was not correlated with the foveal curvature, suggesting that the inner retinal factors described above might be an additional determinant of the MTM development.

Regarding the mCNV, a high association between lacquer crack and mCNV in high myopic eyes suggests a role of mechanical stress and crack at the level of the RPE or the Bruch membrane in mCNV development^20^. Mechanical stretching of the RPE *in vitro* can potentiate the production of vascular endothelial growth factor^21^. In addition, reduced choroidal blood flow may result in a hypoxic state of the outer retina, leading to increased secretion of vascular endothelial growth factor^22^. Ikuno *et al*.^13^ reported that eyes with mCNV had a significantly thinner subfoveal choroid than unaffected fellow eyes with similar axial lengths. In eyes with lower foveal curvature, under the assumption of multidirectional expansion of the staphyloma as per our hypothesis, extreme choroidal thinning may have occurred over the larger area of macula compared to those with greater curvature, resulting in a higher risk of mCNV.

The inferior and temporal staphyloma heights showed no significant difference among the three groups. The reason for this result is unclear, but may be related to the pathogenic mechanism of the optic disc tilting associated with myopic progression^23^. In a previous study, the location and properties of the deepest point of the eyeball differed significantly between the myopic and emmetropic eyes^24^. Most of the deepest points of the eyeball were at the inferior part of the globe in myopic eyes but at the optic disc in emmetropic controls. A further prospective study is required to clarify if the dynamic change in posterior staphyloma is asymmetrical and accompanied by an expansion in the inferotemporal direction of the optic disc.

This study has several limitations. First, the 6-mm scan length employed in this study, although it covered ranges of longer than 7 mm in actual dimension in most cases, was not long enough to cover the entire extent of the staphyloma. Wakazono *et al*.^25^ reported a significant difference in the curvature at 3 to 6 mm from the fovea between high myopic eyes with and without MTM, and staphyloma curvature assessment including this area may provide more insights regarding the mechanism underlying the development of myopic macular complication. Second, this study was cross-sectional in design, and longitudinal progression of MTM and its risk factors could not be evaluated properly. Third, displayed images on B-scan OCT, even after transformation into 1:1 µm mode, do not faithfully represent the true eyeball shape, because sector-scanned images are transformed into rectangles^26^. The measured curvature of staphyloma based on OCT images might be less steep compared to the original shape, but the difference in curvature observed in the MTM and other groups in the present study might not be changed. Lastly, the foveal curvature was measured using manual rather than automated techniques, which could have led to inaccurate measurement and imprecise calculations.

In summary, our finding of different foveal curvatures in MTM patients from those in mCNV or control group with comparably severe high myopia suggests that each myopic macular complication has a different underlying mechanical pathogenesis, and that the elongation of the axial length may not be the sole contributing factor. In addition, we showed that an easily applicable parameter for staphyloma steepness, the curvature index, has a good correlation with known methods for the measurement of foveal curvature. In the progression of pathologic myopia, the foveal curvature may have a predictive value for the development of different types of myopic macular complications. Prospective studies with longitudinal observation of the foveal curvature within posterior staphyloma might provide more insights into the pathogenesis of myopic macular complications.

## Methods

### Participants

In this comparative study, we performed a cross-sectional assessment of the foveal curvature in high myopic patients who have MTM or mCNV within the posterior staphyloma using spectral domain OCT. The protocol and study design were approved by the Institutional Review Board of Seoul National University Hospital (IRB no. 1707-168-873) and were performed in accordance with the tenets of the Declaration of Helsinki. Informed consent was obtained from each participant before study inclusion.

The medical records of patients with high myopic eyes (axial length ≥ 26.5 mm or myopic refractive error ≥ −6.0) who were examined at the Retina Center of Seoul National University Hospital from January 2013 to December 2016 were reviewed. Among the high myopic patients with posterior staphyloma involving the macula, eyes with MTM (MTM group) or mCNV (mCNV group) were included in this study. High myopic patients with macula-involving posterior staphyloma, but without MTM and mCNV, were included as the control group. The presence of posterior staphyloma was determined when characteristic localized outpouching of the posterior pole and its border with surrounding curvature were identifiable with the ultra-wide field images or B-scan ultrasonography (Fig. 1). The diagnosis of MTM was made when abnormal findings including retinoschisis, foveal retinal detachment, lamellar or full-thickness macular hole with or without macular detachment were observed in the spectral domain OCT images^17^. Myopic CNV was diagnosed when a hyperreflective lesion was detected on OCT images in the area corresponding to the hyperfluorescent lesion with late leakage on fluorescein angiography. For all groups, only one eye per patient was included. If both eyes were eligible, only the right eye was included. The spectral domain OCT images taken at the first detection of MTM or mCNV were used for analysis.

The exclusion criteria were as follows: (1) presence of other exudative macular diseases such as age-related macular degeneration, chronic serous chorioretinopathy, and macular edema secondary to retinal vascular diseases; (2) previous history of surgery with extraocular explant such as buckle or encircling, which can influence the curvature of the posterior segment of the eyeball; (3) presence of both MTM and mCNV in one eye; (4) posterior staphyloma not involving the fovea; (5) any previous treatment history for MTM or mCNV, including vitrectomy and intravitreal anti-vascular endothelial growth factor injection; (6) poor OCT image quality due to a dense cataract; or (7) dome-shaped macula. The diagnosis of a dome-shaped macula was based on the presence of an inward bulge of the macular RPE of > 50 µm above a presumed line tangent to the RPE line at the bottom of the posterior staphyloma^27^.

### Examination and curvature measurements

All patients underwent a comprehensive ophthalmic examination including BCVA, slit lamp examination, fundus examination, refractive error (spherical equivalent), axial length measurement, spectral domain OCT examination (Cirrus HD-OCT; Carl Zeiss Meditec, Dublin, CA), and ultra-wide field retinal imaging (Optos 200TX; Optos PLC, Scotland, UK). The axial length was measured with ocular biometry (IOLMaster; Carl Zeiss Meditec, Jena, Germany). The type of posterior staphyloma was determined according to the classification by Ohno-Matsui^5^, which classified posterior staphyloma into 5 types (wide macular, narrow macular, peripapillary, nasal, and inferior) and others, according to its location and extent observed in the fundus examination and ultra-wide field images. According to the META-PM Study classification, coexisting myopic macular changes were classified into 4 categories: category 1, tessellated fundus; category 2, diffuse chorioretinal atrophy; category 3, patchy chorioretinal atrophy; and category 4, macular atrophy^4^.

To measure the foveal curvature, usual spectral domain OCT images with a 1:1 pixel mode were converted to the 1:1 micrometer mode for greater accuracy. In addition, because the lengths in OCT images are affected by the long axial length in patients with high myopia, the actual size on the OCT image was adjusted using a previously reported formula: *t* = 3.382 × 0.01306 × (axial length – 1.82) × *s*, where *t* and *s* represent the actual dimensions and measurements on the OCT image, respectively^28^. Two independent retinal specialists (UCP and DJM) measured the foveal curvature and other parameters listed below, which were then averaged for statistical analysis. Image analysis was performed using ImageJ public domain software (Version 1.47, National Institute of Health, Bethesda, MD, USA; available at http://imagej.nih.ogv/ij/). The subfoveal choroidal thickness (SCT) was defined as the perpendicular distance from the outer edge of the hyperreflective line of the RPE to the choroid-scleral junction at the fovea, and the measurements from vertical and horizontal B-scan images including the fovea were averaged.

The foveal curvature was measured by two known methods, namely staphyloma heights and coefficient *a*, and a novel method, a curvature index, based on the shape of the hyperreflective line of the RPE and Bruch membrane complex in OCT B-scan images including the fovea. All parameters were compared among the MTM, mCNV, and control group. In addition, correlations between the foveal curvature parameters were evaluated.

**Figure 4:**
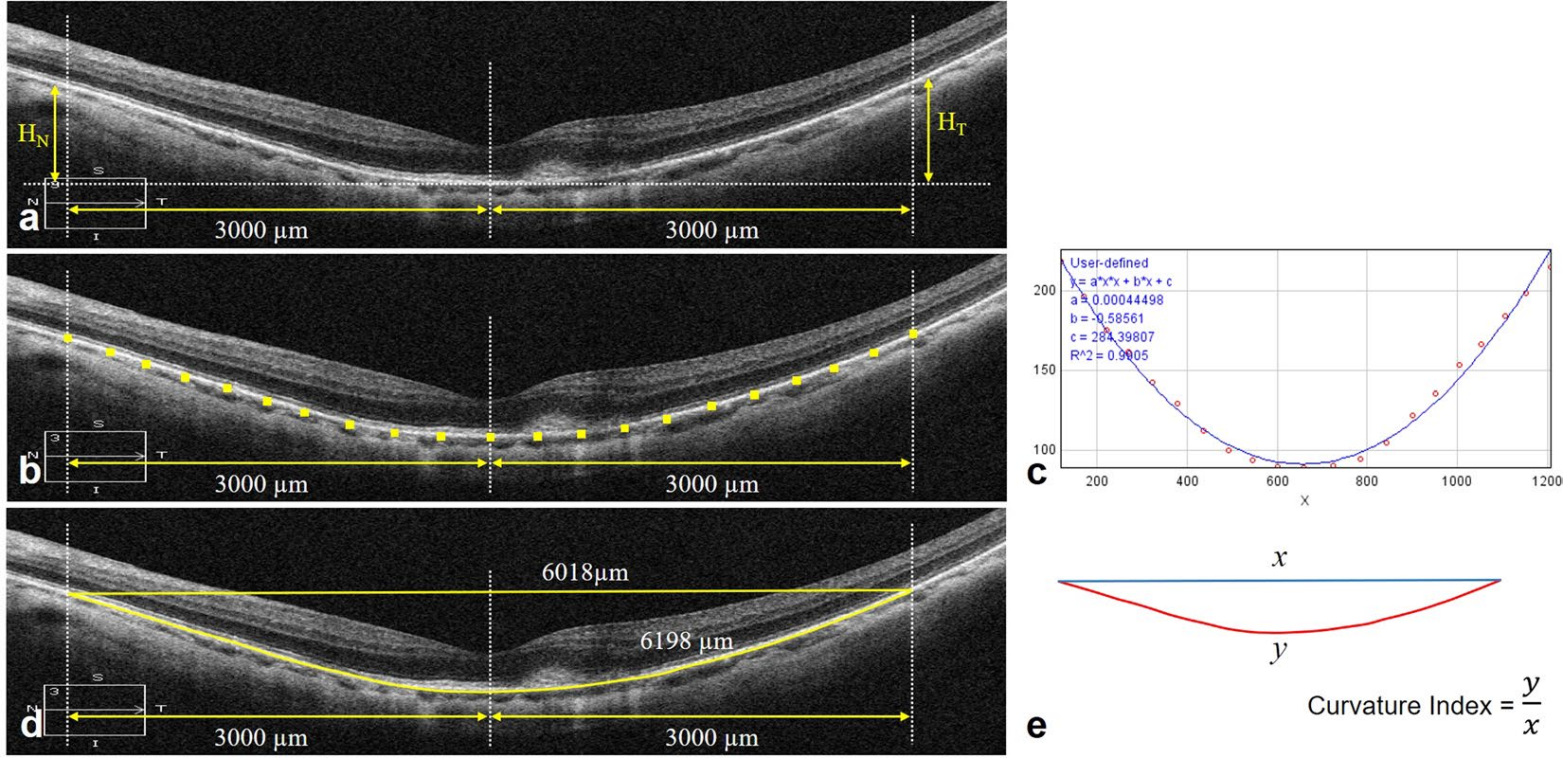
Measurement of the posterior staphyloma curvature using optical coherence tomography B-scan images converted to 1:1 μm mode. (**a**) The staphyloma height was measured as the vertical distance from the retinal pigment epithelium (RPE) line at 3 mm nasal, temporal, superior, and inferior from the fovea to the level of the subfoveal RPE. (**b**,**c**) For coefficient *a*, points at 300-μm intervals along the 6-mm RPE line were exported to ImageJ. A second-order polynomial equation (*ax*^2^ + *bx* + *c*) best fit with the plots was determined; coefficient *a* (4.45 × 10^−4^) represents the steepness of the RPE curve. (**d**,**e**) For the curvature index, the length of the RPE line between two points at 3-mm distance from the fovea was divided by a straight distance between the two points (6198 μm/6018 μm = 1.030).

The staphyloma height was measured as the vertical distance from the peripheral RPE line at 3 mm nasal, temporal, superior, and inferior from the fovea to the level of the subfoveal RPE using vertical and horizontal B-scan OCT images including the fovea (Fig. 4a)^13^. It was recorded as a negative value when the peripheral RPE/Bruch membrane was located posterior to the subfoveal RPE. A total staphyloma height was defined as the sum of the nasal, temporal, superior, and inferior heights, while the horizontal and vertical heights were defined as the sum of the nasal and temporal heights and the sum of the superior and inferior heights, respectively. For coefficient *a*, plots with intervals of 300 μm along the RPE line on the OCT image were exported to ImageJ. A second-order polynomial equation (*ax*^2^ + *bx* + *c*) best fit with the plots was determined using the ImageJ curve-fitting program (Fig. 4b,c); coefficient *a* represents the steepness of the RPE curve^12^. To determine the curvature index, we measured the length of the RPE line between two points at a 3-mm distance from the fovea (6 mm apart) on a B-scan image including the fovea, and the RPE length was divided by the straight distance between the two points to yield a curvature index (Fig. 4d,e). For the coefficient *a* and curvature index, the values obtained using horizontal and vertical B-scan images and their averages were compared among the groups.

In each group, correlations of the foveal curvature parameters with the severity of myopia, the axial length and refractory error, were evaluated. In addition, correlations between the foveal curvature parameters and the severity of each myopic macular complication, which was represented by the FSH for the MTM group and the size of CNV for the mCNV group, respectively, were also evaluated. In the MTM group, FSH was measured manually using a caliper provided by the OCT software as the perpendicular distance between the internal limiting membrane and inner edge of the hyperreflective line of the RPE at the foveal center. In MTM cases with a full-thickness macular hole, the imaginary line of the internal limiting membrane connecting both edges of the hole was used to measure FSH. In the mCNV group, the area of the CNV was described by the disc area, as a unit, based on the digitalized fluorescein angiography images obtained in the early phase.

### Statistical analysis

The demographic and clinical characteristics were compared among the three groups using the chi-square test or one-way analysis of variance as appropriate. For continuous variables, *post-hoc* comparison between each two groups was performed using t-test with Bonferroni’s method to correct for multiple comparisons. Associations between each method for curvature measurement and between the curvature and severity of each myopic macular complication were assessed using correlation analyses. Statistical analyses were performed using SPSS version 22.0 (IBM Corp., Armonk, NY, USA); *P* values < 0.05 were considered significant.

## Supplementary information

Supplementary Figure S1

## Data Availability

The datasets generated during and/or analyzed during the current study are available from the corresponding author on reasonable request.
