# Supplementary material for: Influence of the foveal curvature on myopic macular complications
Source: Sci Rep. 2019 Nov 15;9:16936. doi: 10.1038/s41598-019-53443-4 (PMC6858376; doi:10.1038/s41598-019-53443-4)
Supplement: Supplementary file 1 — Supplementary Figure S1 [file 41598_2019_53443_MOESM1_ESM.pdf]

Title: Influence of the foveal curvature on myopic macular complications

Authors

Un Chul Park[1,2], Dae Joong Ma[1,2], Woon Hyung Ghim[1,2], Hyeong Gon Yu[1,2]

[1]Department of Ophthalmology, Seoul National University College of Medicine, Seoul, Korea

[2]Retinal Degeneration Research Laboratory, Seoul National University Hospital Biomedical Research Institute, Seoul, Korea

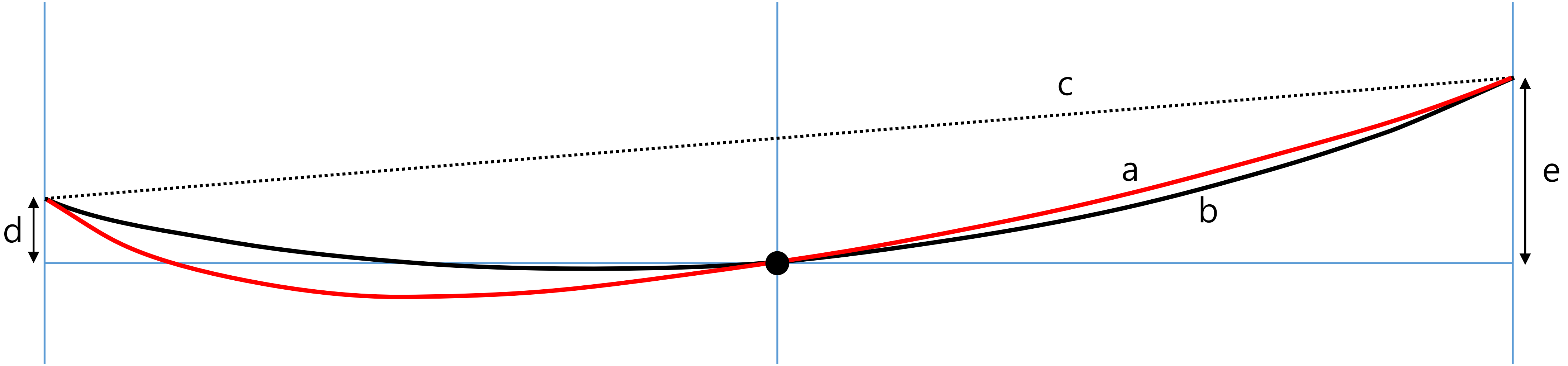

Supplement Figure. A diagram showing usefulness of the curvature index as a measurement for posterior staphyloma curvature. Although RPE lines of black and red color have same staphyloma heights (d, e) at two points at 3-mm distance from the fovea (black dot), curvature index of the red line (a / c = 1.084) is greater than that of black line (b / c = 1.048) showing more prominent posterior bowing. This shows that the curvature index is more representative of the staphyloma contour than the staphyloma height especially in eyes with asymmetric contour of staphyloma which has the deepest point not at the fovea.
